# Academic accountability to local communities and society through Programme Science: a case study from the HPV self‐sampling programme HOPE in Peru

**DOI:** 10.1002/jia2.26297

**Published:** 2024-07-10

**Authors:** Patricia Jannet Garcia, Carlos Santos, Marina Chiappe, María Valderrama, Cesar Paul Carcamo

**Affiliations:** ^1^ School of Public Health Cayetano Heredia University Lima Peru; ^2^ Directorate of Cancer Prevention and Control Ministry of Health Lima Peru

**Keywords:** academic accountability, cervical cancer, HPV, Peru, Programme Science, women

## Abstract

**Introduction:**

Health challenges in the 21st century underscore the need for adaptable and innovative approaches in public health. Academic institutions can and should contribute much more effectively to generate and translate scientific knowledge that will result in better programmes to improve societal health. Academic accountability to local communities and society requires universities to actively engage with local communities, understanding the context, their needs, and leveraging their knowledge and local experience. The Programme Science initiative provides a framework to optimize the scale, quality and impact of public health programmes, by integrating diverse approaches during the iterative cycle of research and practice within the strategic planning, programme implementation and programme management and evaluation. We illustrate how the Programme Science framework could be a useful tool for academic institutions to accomplish accountability to local communities and society through the experience of Project HOPE in Peru.

**Discussion:**

Project HOPE applied the Programme Science framework to introduce HPV self‐sampling into a women's health programme in Peru. Collaboration with local authorities and community members was pivotal in all phases of the project, ensuring interventions aligned with community needs and addressing social determinants of health. The HOPE Ladies—community women trained and empowered to promote and provide the HPV kits—crafted the messages used through the study and developed strategies to reach individuals and provided support to women's journey through health centres. By engaging communities in co‐creating knowledge and addressing health inequities, academic institutions can generate contextually relevant and socially just scientific knowledge. The active participation of community women in Project HOPE was instrumental in improving service utilization and addressing barriers to self‐sampling.

**Conclusions:**

The Programme Science approach offers a pathway for academic institutions to enhance their accountability to communities and society at large. By embedding researchers within public health programmes and prioritizing community engagement, academic institutions can ensure that research findings directly inform policy improvements and programmatic decisions. However, achieving this requires a realignment of research agendas and recognition of the value of community engagement. Establishing Programme Science networks involving academia, government and funding entities can further reinforce academic accountability and enhance the impact of public health programmes.

## 1 INTRODUCTION

Health issues in the 21st century are increasingly complex. Populations worldwide are expanding and ageing, leading to a rise in non‐communicable diseases added to the emerging and re‐emerging infections which remain a major public health issue [1, 2]. To address these global health challenges, we must adapt health systems to prioritize prevention and promote investments and advancements in innovation and research leading to better access to effective care.

Universities play a pivotal role in advancing knowledge. In this regard, academic institutions can and should contribute much more effectively to generate and translate scientific knowledge that will result in better programmes to improve societal health. However, in the past decades, the way academic institutions interact with society has been challenged, prompting a call for a “new social contract” to ensure scientific knowledge is both robust and transparent, involving society in its production. Concepts like “socially robust scientific knowledge” and “academic accountability to local communities and society” have entered the discourse as a result [3, 4]. This shift demands universities go beyond traditional academic measures, becoming more accountable to society by actively engaging with local communities, understanding their needs, the context, tapping into community expertise, and leveraging academic and research capabilities to address challenges effectively and result in positive impacts [3]. Achieving academic accountability may not be an easy task, since it involves the complex interplay of multiple factors such as institutional culture, leadership and resources, between others.

Programme Science was initially introduced and applied in the context of HIV, sexually transmitted infections and blood‐borne infections, and more recently, it has been extended to maternal health as well [5, 6, 7]. This approach seeks to bridge the gap between research and practice in public health by employing an iterative, multiphase research cycle that includes three key spheres: strategic planning, programme implementation, and programme management and evaluation, so that practice informs research and research informs practice and policy [8]. The Programme Science framework integrates and expands upon various research frameworks such as implementation science, operations research and translational research. It advocates for diverse approaches to gather essential information, adapt interventions and conduct real‐time evaluations within evolving public health programmes promoting community engagement and mobilization and context‐specific adaptations [9, 10]. We aim here to illustrate, with a case study from Peru, how Programme Science, this new framework to optimize the scale, quality and impact of public health programmes, could be a useful tool for academic institutions to accomplish accountability to society.

## 2 DISCUSSION

We built upon and adapted the Programme Science framework to apply it to a feasibility study of the introduction of HPV self‐sampling into a regional women's health programme in Peru: Project HOPE [11]. Precisely, during the phases of strategic planning, programme implementation and programme management and evaluation, we worked closely with local authorities and engaged women from the community. Together, we assessed the context and local constraints, co‐developed the intervention package, defined where and how to deliver the intervention for success and collected data which was eventually used for further improvements in the programme translating the research findings into practice and policies.

Programme Science emphasizes the importance of collaboration among researchers, decision‐makers, implementers and community members. Academic institutions can actively engage with these stakeholders, facilitating partnerships, as they are seen as natural and neutral conveners. Collaborative approaches foster shared decision‐making, build trust, promote ownership, and enhance the relevance and sustainability of the work which could result in better public health outcomes and benefits for communities. Following this principle, we established collaborations with the local authorities since the design of the study. The Programme Science framework highlights the importance of understanding the populations we are working with, and their changing needs as well as defining the mix of interventions to be implemented, and where and how to best deliver them. This requires engaging community members as active participants in the research process [9]. This process can ensure that academic work addresses real‐world challenges and contributes to community empowerment. This can be achieved through different methodologies, for example participatory research methods, community‐based participatory research or community advisory boards. Community engagement can also allow academic institutions to develop research agendas that are informed by and responsive to questions arising within programmatic and community contexts.

In our study design, we did not just involve the community; we also fostered opportunities for co‐creating knowledge. Programme Science emphasizes the importance of addressing social determinants of health and health inequities. By incorporating a social justice perspective into research and programme development, academic institutions can play a role in mitigating health disparities and fostering fair health outcomes within communities. In our study, we aimed to tackle inequities by providing alternatives to reach women who might face barriers in accessing healthcare services.

Project HOPE was conducted in the district of Ventanilla in the region of Callao, east of Lima, the capital of Peru, between 2015 and 2016. The authorities of the Health Directorate of Callao (DIRESA Callao) identified Ventanilla as the area with the highest number of cases of cervical cancer in their region. Most people in the area are migrants from the rainforest and Andean regions of the country where cervical cancer screening rates are low. Together with the local authorities, we mapped the Ventanilla health clinics’ networks and the resources available and discussed the study design. The study was deemed to be a 1‐year feasibility study with the aim of reaching around 2000 women from the community to evaluate the acceptability of HPV self‐sampling, satisfaction of the users, efficiency of the distribution of kits by women in the community, feasibility of performing the testing locally and documenting the prevalence of HPV positivity and the performance of the HPV test (sensitivity and specificity) in local conditions. To engage women from the community in the study, we placed posters at markets, streets, community centres and schools. The objective was to recruit adult women (18 years or older) living in Ventanilla, who would like to volunteer to promote HPV self‐sampling, helping other women in their community. We interviewed 108 women who were interested and invited 59 women to the training sessions, mainly those who had the desire and availability to dedicate at least a couple of hours three times a week to the study. The interviews provided valuable insights into women's knowledge, emotions, concerns and suggestions regarding cervical cancer and health services. This information guided the content of the 2‐day training workshop, held at the community centre. Using participatory techniques and role‐playing, women learned about cervical cancer, HPV, HPV self‐sampling, data recording and worked on abilities for effective communication. As part of the training, all the participants had their own experience with self‐sampling and got their own HPV results.

One important discussion during the training was how and where to approach women from the community to start talking about something as sensitive as cervical cancer or vaginal samples, and participants contributed with their experiences to create the “script” to approach other women. They also suggested the benefit of having a corporate look and adopted the name “HOPE Ladies.” Most importantly, since a recurring theme was that most women encounters were at the markets, where market bags are needed, women suggested designing a market bag where all the HPV self‐sampling kits and educational materials could be carried, that should look “normal” on the outside, but that could be opened to show the external and internal female reproductive organs so that volunteers could explain to other women how they looked from the inside and how to collect their own vaginal samples. We then started the process of co‐creation which resulted in the HOPE bag which facilitated and improved understanding of self‐sampling, and eased communication between the HOPE Ladies and the women in the community.

After the training, the HOPE Ladies received 10 kits (careHPV Sample Collection and care brush) to be offered to women 25−59 years old and gathered participant data, labelled kits and instructed home sample collection. Participants placed labelled kits in provided mailboxes at health centres. Initially, weekly meetings were held between the HOPE Ladies and the project team to report distribution and address issues. Meetings later became monthly, focusing on improving HPV kit acceptance and distribution.

With local authorities, the project team established biweekly motorcycle transportation to transfer samples from mailboxes to the DIRESA Callao Laboratory for processing. HPV results, which were entered into a database, and SMS notifications were sent to participants. Ninety percent of women with negative HPV tests received SMS results and recommendations; the rest had clinic appointments scheduled. All women with positive results received clinic appointments via SMS.

At the clinic, women were briefed on study procedures, including medical examination, PAP smear, colposcopy, biopsies and treatment if needed, and asked for consent to participate. Cryotherapy treatment was provided to women during the same visit if lesions were visible, regardless of HPV status. Biopsies were taken if lesions were present or as per protocol. PAP smears and biopsies were sent to Lima for analysis. Women returned for a second visit to receive pathology results, and those requiring treatment received cryotherapy at the primary care centre or were referred for further management. During the 20‐week period of the study, 2217 HPV sampling kits were distributed. About 95% (2112) of kits arrived in good condition at the laboratory for testing, with 22 duplicate samples, totalling 2090 women tested. Twelve women were excluded for being outside the testing age range. Out of 2078 women finally included in the analysis, 266 (12.8%) tested positive for HPV. Two‐thirds of women who provided a self‐sample for HPV testing reported never having a PAP smear.

Around 98% (2034) of women provided a cell number for SMS results; only 0.8% were incorrect. Besides SMS, HOPE Ladies gave result envelopes. All 266 HPV‐positive and 180 HPV‐negative women received follow‐up appointments, with reminders. Most attended in a timely manner (98% HPV positive, 98% HPV negative). Home visits were made to the six women HPV‐positive no‐shows. Eight HPV‐positive and 14 HPV‐negative women did not consent to continue in the study, but they received result counselling and were managed accordingly. All consenting HPV‐positive women (252, 95%) and 160 HPV‐negative (89%) completed procedures. Thirty‐six women (8.2%) had high‐grade lesions or cancer; 34 were HPV positive (Table 1).

**Table 1:** Results from colposcopy and biopsies for women with positive and negative human papilloma virus (HPV (+), HPV (−)) molecular test results

	HPV (+)	HPV (−)
	*N* (%)	*N* (%)
**Colposcopy results**	252	160
No precancer lesion^a^	193 (76.6)	141 (88.1)
Low‐grade lesion	35 (13.9)	13 (8.1)
High‐grade lesion	22 (8.7)	4 (2.5)
Cancer	0	0
No visualization of cervix	2 (0.8)	2 (1.2)
**Biopsy results**	252	160
No precancer lesion^a^	132 (52.4)	115 (71.8)
Low‐grade lesion	77 (30.5)	39 (24.3)
High‐grade lesion	32 (12.7)	2 (1.2)
Cancer	2 (0.8)	0
Inadequate/insufficient sample	9 (3.6)^b^	4 (2.5)^b^

Abbreviation: HPV, human papillomavirus.

^a^ Includes normal, cervicitis, atrophy, others.

^b^ Results from colposcopy and PAP smear test for these cases showed low‐grade lesions or cervicitis. All patients were followed up and received management accordingly.

The sensitivity and specificity of HPV testing for high‐grade lesions were 63% and 88%, respectively (the estimated sensitivity of PAP cytology in Peru ranges between 30% and 35%) [13, 14]. Thirty‐seven women with high‐grade lesions received cryotherapy; nine were referred.

A survey and interviews showed high satisfaction with the programme (75%) and a preference for self‐sampling (70%), consistent with existing literature findings [14]. Women praised the HOPE Ladies’ involvement, since they “make them feel more comfortable.” Most of the interviewed women (96.9%) said they would participate in the programme again and 99% would recommend it to their family and friends [11].

In this project HOPE, the active participation of community women was crucial in addressing disparities and barriers to the introduction of HPV self‐sampling and improving service utilization. The HOPE Ladies offered insights on reaching difficult‐to‐access individuals, crafted messages and provided support throughout the women's journey through the health centres. Delivering test results via SMS initially appeared to be an innovative and effective approach. However, during follow‐up in‐person visits to deliver envelopes with results, as was required by the health centre, we found that only 12% of women acknowledged receipt of the SMS due to connectivity issues and SMS overload. Collaboration with health authorities enabled changes in screening processes, including sample collection, transportation and dedicated clinics. Our study was instrumental in promoting changes in the National Guidelines for Cervical Cancer Screening in Peru, which were released in 2017, and include the use of HPV molecular testing and self‐sampling. The implementation process has been gradual, and we are still at the beginning stages in the country. The HOPE project results contributed to the expanding global evidence indicating that HPV self‐sampling can enhance cervical cancer screening participation compared to the standard of care. However, a recent systematic review also underscored the minimal impact that HPV self‐sampling has on linking individuals to clinical assessment/treatment, presenting the next programmatic challenge to address [15]. Currently, we are working on a more comprehensive model for cervical cancer screening and management in the Andean Region of Peru. This model incorporates not only self‐sampling, but also new technologies aimed at addressing barriers to effective treatment within the existing healthcare system.

## 3 CONCLUSIONS

The Programme Science approach provides a framework for academic institutions to offer more relevant and timely research to enhance public health programmes and to be accountable to communities [16, 17, 18]. Embedding academic researchers within key public health programmes, as exemplified in the HOPE project, ensures alignment between research and the knowledge needs of programmes, policymakers and society. Collaboration, community engagement, contextualization, co‐creation of knowledge during the cycle of strategic planning, implementation and programme management and evaluation are critical steps to facilitate more efficient generation and translation of knowledge to inform policy improvements and to optimize the scale, quality, and impact of public health programmes. However, achieving this requires academic institutions (and funding bodies) to realign research agendas to focus on immediate programme knowledge needs. Academic institutions must recognize the value of such engagement and adjust incentive structures accordingly to support researchers embedded within programme contexts. Moreover, establishing Programme Science networks involving aligned academic, governmental and funding entities would not only bolster academia's impact but also reinforce academic accountability to local communities and societies.

## COMPETING INTERESTS

The authors declare no competing interests.

## AUTHORS’ CONTRIBUTIONS

Conceptualization: PJG. Field work: CS, MC and MV. Data analysis: PJG and CPC. Writing original draft: PJG. Writing review and editing: PJG, CS, MC, MV and CPC.

## FUNDING

None to write the article, but the HOPE project was financed by Grand Challenges Canada, Stars in GH Round 7 Phase 1 #0686‐01‐10.

## Data Availability

Data sharing is not applicable to this article as no datasets were generated or analysed during the current study.
